# Genetic markers of pigmentation are novel risk loci for uveal melanoma

**DOI:** 10.1038/srep31191

**Published:** 2016-08-08

**Authors:** Robert Ferguson, Matjaz Vogelsang, Esma Ucisik-Akkaya, Karan Rai, Robert Pilarski, Carlos N. Martinez, Justin Rendleman, Esther Kazlow, Khagay Nagdimov, Iman Osman, Robert J. Klein, Frederick H. Davidorf, Colleen M. Cebulla, Mohamed H. Abdel-Rahman, Tomas Kirchhoff

**Affiliations:** 1 Perlmutter Cancer Center, New York University School of Medicine, New York, USA; 2 Departments of Population Health and Environmental Medicine, New York University School of Medicine, New York, USA; 3 The Interdisciplinary Melanoma Cooperative Group, New York University School of Medicine, New York, USA; 4 Division of Human Genetics, Department of Internal Medicine, The Ohio State University, Columbus, OH, USA; 5 Department of Medicine, New York University School of Medicine, New York, USA; 6 Ronald O. Perelman, Department of Dermatology, New York University, New York, USA; 7 Department of Genetic and Genomic Sciences, Icahn School of Medicine at Mount Sinai, New York, USA; 8 Havener Eye Institute, Department of Ophthalmology and Visual Science, The Ohio State University, Columbus, OH USA

## Abstract

While the role of genetic risk factors in the etiology of uveal melanoma (UM) has been strongly suggested, the genetic susceptibility to UM is currently vastly unexplored. Due to shared epidemiological risk factors between cutaneous melanoma (CM) and UM, in this study we have selected 28 SNPs identified as risk variants in previous genome-wide association studies on CM or CM-related host phenotypes (such as pigmentation and eye color) and tested them for association with UM risk. By logistic regression analysis of 272 UM cases and 1782 controls using an additive model, we identified five variants significantly associated with UM risk, all passing adjustment for multiple testing. The three most significantly associated variants rs12913832 (OR = 0.529, 95% CI 0.415–0.673; p = 8.47E-08), rs1129038 (OR = 0.533, 95% CI 0.419–0.678; p = 1.19E-07) and rs916977 (OR = 0.465, 95% CI 0.339–0.637; p = 3.04E-07) are correlated (r^2^ > 0.5) and map at 15q12 in the region of *HERC2/OCA2*, which determines eye-color in the human population. Our data provides first evidence that the genetic factors associated with pigmentation traits are risk loci of UM susceptibility.

Uveal melanoma (UM) is the most common primary adult intraocular cancer^1^,^2^ with relatively unclear etiology, although some specific risk factors, such as ethnicity or eye/skin pigmentation traits, have been suggested^3^. Our previous studies show that about 12% of UM manifest as a highly penetrant familial syndrome, often involving a variety of other cancers including cutaneous melanoma (CM)^4^,^5^, suggesting that genetic susceptibility likely plays an important role in the etiology of UM. Despite this, currently known highly penetrant germline mutations in *BAP1, CDKN2A*, or *BRCA2* explain only about 3% of UM population-specific risk^6^,^7^,^8^,^9^. Thus, developing clinically relevant UM risk prediction models that account for both genetic and host factors is currently difficult, also because of a vastly unexplored role of low-penetrant genetic risk factors in the general UM population. Unlike other solid tumors, no genome-wide association study (GWAS) data exists for UM as large sample cohorts for this rare cancer are generally unavailable. Although CM and UM have distinct somatic genetic characteristics that reflect different melanocytic origin^3^,^10^,^11^, the co-occurrence of CM and UM in a subset of affected families suggests a shared predisposition to both cancer types^5^. Recently, a number of genetic variants have been reproducibly associated in GWASs with the risk of CM and skin/eye pigmentation traits (Supplementary Table 1). The shared etiological risk factors between CM and UM (including pigmentation) suggest that a subset of CM risk variants would associate with genetic susceptibility to UM.

## Results and Discussion

Herein, we analyzed 28 SNPs (one SNP did not pass genotype filtering) established in recent GWASs for association with CM or related host phenotypes (including skin and eye pigmentation) (Supplementary Table 1), in a population of 272 UM patients and 760 controls that were of European ancestry (Supplementary Table 2). In addition, to increase the statistical power, we have included genotyped data of 1047 controls from a publically available GWAS (phs000187.v1.p1)^12^. Logistic regression analysis adjusted by age and gender revealed a novel association with reduced UM risk in the locus of HERC2/OCA2 at 15q13 for 3 correlated SNPs (r^2^ > 0.5), which were still significant following adjustment for multiple testing (Table 1, full results in Supplementary Table 3): rs12913832 (OR = 0.529, 95% CI 0.415–0.673; p = 8.47E-08), rs1129038 (OR = 0.533, 95% CI 0.419–0.678; p = 1.19E-07) and rs916977 (OR = 0.465, 95% CI 0.339–0.637; p = 3.04E-07). All three SNPs were previously found to be associated in GWAS with both eye pigmentation^13^ and CM risk^12^,^14^. Similarly, the strongest effect has been observed in both phenotypes for rs12913832. The directionality and magnitude of odds ratios for all three SNPs reported for CM and UM risk are similar (for rs12913832 OR = 0.69 versus 0.53, respectively), further suggesting that pigmentation is a shared etiological risk factor between both diseases. The associations with UM risk remained comparably significant after adjustment for family history of other cancers (including family history of CM), personal history of CM or major UM subtypes (for rs12913832: p = 7.08E-06, p = 1.23E-06 and p = 3.06E-06, respectively). To further test the robustness of these findings we also performed association analysis of Ohio State University Medical Center (OSUMC) cases against other independent control populations from recent GWASs that were publically available (Supplementary Table 4). As shown, the associations remained comparably significant for each control set as well as in the pooled aggregate analysis.

The locus at 15q13.1 determines pigmentation of eyes and skin by regulating the expression of *OCA2*, which codes for *P* protein involved in melanin synthesis^15^. Prior functional data show that our most significant variant rs12913832 in the intronic region of *HERC2*, is a key pigmentation “regulator allele” that impacts the expression of *OCA2* via a long range enhancer mechanism^16^. The less common T-allele (darker eye color) of rs12913832, associated with UM-protective effect in our data, enhances *OCA2* expression resulting in darkly pigmented melanocytes. The C-allele conversely reduces expression of *OCA2* producing lightly colored melanocytes^16^. Taking this functional data together with our findings suggests that genetic determinants of dark eye pigmentation are protective while lighter pigmentation alleles confer a risk effect on UM^3^,^17^. Importantly, these genetic observations are also in clear alignment with previous epidemiological studies demonstrating that light eye color is indeed a UM risk factor (OR = 1.75 95% CI 1.31–2.34 p = <0.001)^18^.

**Figure 1:**
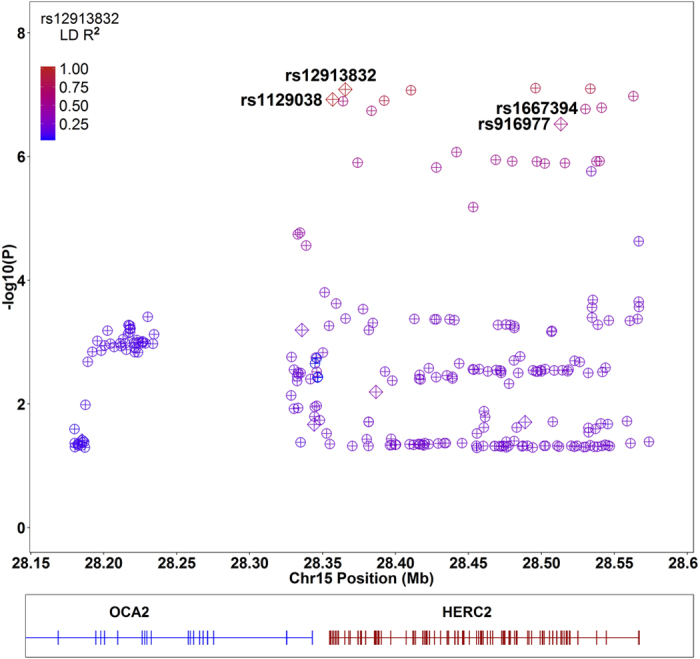
The regional plot of associations at HERC2/OCA2 locus with UM risk. The most significant associations at the HERC2/OCA2 locus identified in this study were analyzed using imputation-based fine mapping. The genotyped variants (diamonds) were imputed (circles) with an INFO score >0.5. The strength of association with UM risk is displayed as [−log_10_(p)] versus chromosomal position (Mb). Variants are colored according to LD with rs12913832 calulated from 1000 Genomes Project Phase 3 using the data from European ancestry population (N = 503). Associations were noted for imputed variant, rs1667394 (r^2^ = 0.48 with rs12913832), previously reported in a GWAS on eye color^19^.

In an attempt to explore other possible genetic loci with UM-specific regulatory function, we have also performed an imputation analysis of associations at the HERC2/OCA2 region. While we found no other association surpassing the effect of rs12913832 (Fig. 1, Table 1 and Supplementary Table 3), interestingly, imputed data showed an association signal, albeit with reduced significance, for another correlated variant, rs1667394 (r^2^ = 0.48) (Supplementary Table 5), that was also previously identified in a GWAS for association with eye color^19^,^20^. While the data with imputed variants confine the association locus to a narrow ~250 kb region at HERC2/OCA2, none of the associations remained statistically significant after conditioning the analysis for rs12913832 (data not shown). This suggests that all observed associations at HERC2/OCA2 stem from a signal driven by rs12913832. These findings together with prior established functional data on rs12913832 and eye pigmentation^16^ indicate that this is the strongest candidate to be biologically relevant in UM development. There is, however, a large degree of correlation among the associated genotyped variants that served as input for imputation analysis at HERC2/OCA2. As such, more focused physical fine-mapping in this locus needs to be performed to rule out the presence of additional biologically impactful UM-specific loci that are independent of rs12913832.

It is important to note that our study has limited power and hence may reduce the ability to detect associations exerting smaller risk effects. It is possible that there are other associations among the selected variants that are likely missed in this study due to relatively small size of our UM case population. The most significant associations at HERC2/OCA2 locus (Table 1), as well as rs12203592 in IRF4, all show odds ratios <0.6 or >1.4, respectively (Table 1). As we estimated, to detect such risk effect magnitude given the current case/control sample size, our study has >95% power (under MAF>0.05, alpha = 8.9E-04). However, the power rapidly drops for the expected effect size of OR < 1.3 (approximately 40% power reduction). Hence, to provide a true estimate of potential risk effect with smaller size for remaining subset of variants in our selection, a larger study will be needed as part of national or international consortia. As part of such collaborative efforts, the consideration of additional covariates such as pigmentation phenotypes in studied populations would be essential, as in the current study, while important, this data was not available.

Also, the relatively small size of our case population did not allow for stratifying the associations by the primary location of tumors, and this will need to be explored in subsequent large consortia analyses. The molecular effect of pigmentation on different UM subtypes has recently been suggested, as different *GNAQ* mutation signatures were observed in posterior (choroid) versus anterior UM tumors (ciliary body, iris), likely reflecting divergent UM pathways related to toxic pheomelanin synthesis^17^. This molecular support for a role of melanin in UM histologies and the association of germline variants of eye color with UM risk found in this study suggest interplay between both somatic and inherited factors of pigmentation pathways in UM development.

A limitation in this study is an inability to assess the effect of pigmentation phenotype information in our sample populations as the HERC2/OCA2 locus exerts a purifying selection effect in European ancestries^21^,^22^. Our analysis employed a set of control subjects matched by geographical location, age and gender to the cases, ascertained at OSUMC and an additional US control population of white ancestry (ascertained at MD Anderson). Both control sets show comparable allele frequencies of HERC2/OCA2 variants, indicating similarities in distribution of pigmentation in both control sets. Also, it has been previously demonstrated that the distribution of pigmentation variants, including rs12913832, in European populations along a north-south axis often manifests with significant Hardy Weinberg equilibrium (HWE) departures, likely as reult of underlying selection pressure for these alleles^22^. Although the HWE test does not inform on the population stratification, the fact that these pigmentation alleles are in HWE in both control sets used here (separately or in aggregate) further suggests that misbalanced distribution of pigmentation traits potentially confounding our findings, is unlikely.

The identification of novel germline genetic loci involved in UM susceptibility in our study provides the first evidence of a link between the inherited genetics of pigmentation and UM risk. It has been established that lighter pigmentation and chronic sun exposure impact the development of choroid nevi, which occur in ~7% of the US population and are a known precursor for UM^23^. Testing the associations in this study in the context of UM risk and the presence of ocular nevi will also be important in future analyses. Complementing our findings with other high-risk loci (e.g. BAP1) will further expand the clinical implications for both UM risk-prediction and biological understanding of pigmentation in UM etiology.

## Methods

### Study population

For cases, the study originally employed 286 UM patients. Fourteen of these failed the genotyping or quality control, resulting in 272 UM cases available for association analysis. The vast majority of patients were ascertained from the ophthalmology, medical oncology or clinical cancer genetics clinics at Ohio State University. Four patients were referred to our research program from an outside institute. UM patients with family history of UM were excluded. The study was approved by The Ohio State University cancer institutional review board. DNA for patients and controls were extracted from peripheral blood lymphocytes by a simple salting out procedure^24^. Seventy-three patients, including two with tumors of the iris, were treated by enucleation and the remaining by local therapy, mostly brachytherapy. Basic demographic data such as age at diagnosis, sex and ethnicity were collected for each patient. Personal history and a three generation family history of cancers were also collected from each patient. Personal history of other cancers was confirmed by medical records and or pathology. Family history of cancers was confirmed whenever possible. Most of the diagnoses in this study represented choroidal or ciliochoroidal UM (n = 253) with a smaller fraction of the iris UM (n = 16). From 272 cases, n = 133 were males [median age 59 (Range: 24–84 years)] and n = 139 were females [median age 59 (Range: 18–84 years)]. The detailed clinical and demographic information is summarized in Supplementary Table 2. Control samples were provided by the OSUMC Human Genetics Sample Bank. The Columbus Area Controls Sample Bank is a collection of control samples for use in human genetics research that includes donors’ anonymized biological specimens and linked phenotypic data. The data and samples are collected under the protocol “Collection and Storage of Controls for Genetics Research Studies”, which is approved by the Biomedical Sciences Institutional Review Board (IRB) at OSUMC. The methods in this study were carried out in accordance with guidelines set forth by IRB at NYUMC relating to the use of patient samples in genetic studies. Recruitment takes place in OSUMC primary care and internal medicine clinics. All individuals included provided written informed consent, completed a questionnaire that includes demographic, medical and family history information, and donated a blood sample.

Out of available specimens from 3500 participants currently ascertained by OSUMC Human Genetic Sample bank, for our analysis we selected 760 controls that were individually matched as closely as possible to cases by gender and age (Supplementary Table 2). The DNA specimens from the controls were obtained from peripheral blood. Twenty five of the 760 controls failed the genotyping or quality controls, leaving 735 controls available for the association analysis. All cases and controls were of European ancestry, and the ethnicity information was determined based on self-reported ancestry. Additional controls, proximal to OSUMC case/control subsets, were used in the study from publically available GWAS data on cutaneous melanoma ascertained at MD Anderson Cancer Center (n = 1047 controls, phs000187.v1.p1)^12^.

### Selection of SNPs and genotyping

To test the association of common genetic variants (MAF > 0.05) with the risk of UM, a total of 29 SNPs were selected through the comprehensive search of published GWAS data on melanoma risk, nevi-driven phenotypes, pigmentation, hair color, skin color and other melanoma risk etiologies. The selection criteria focused on variants with the most significant associations (in particular those surpassing GWAS level of significance) reported from each of these published GWAS. The complete list of selected variants with association information from prior studies is in Supplementary Table 1.

For the genotyping of 29 selected variants, the highly multiplexed Sequenom MassARRAY system (Agena Bioscience Inc, CA) was used according to the manufacturer’s protocol. Quality control (QC) measures included duplicates (8 per each 384-well plate) and non-template controls (2 per plate) resulting in >99% observed concordance with no evidence of cross-contamination. Post-genotyping filtering criteria were as follows: exclusion of SNPs with minor allele frequency (MAF) <5%, exclusion of SNPs with a call rate <95%, exclusion of samples with a call rate <95%, and exclusion of SNPs with significant departure from Hardy-Weinberg equilibrium (p < 0.001). The filtering steps excluded one variant with MAF < 0.05, resulting in genotype information on 28 variants for 272 UM patients and 735 matched controls.

### Statistical analysis

The association of UM risk with the selected 29 common genetic variants in the analysis of 272 cases and 1782 controls was estimated as odds ratios (OR) with 95% confidence intervals (95% CI), using unconditional logistic regression in an additive model where two copies of the minor allele have twice the effect of one copy. The genotype model (separate indicators for heterozygotes and rare homozygotes), dominant model (indicator for heterozygotes and rare homozygotes combined) and recessive model (indicator for homozygotes) were also assessed but not reported as they presented no significant additional information not captured by the additive model. All models were adjusted for continuous age at diagnosis (cases) or at the time of inclusion in the study (controls) and gender. Deviations of the genotype frequencies in the controls from those expected under Hardy-Weinberg equilibrium were evaluated by χ^2^ tests (1 degree of freedom). All analyses were performed in PLINK^25^. For genetic variants not present in publically available controls we have used the nearest proxy (r^2^ = 1) determined from European ancestries in the recent data release of 1000 Genomes Project Phase 3 (October 2014), as indicated in Supplementary Table 3. Throughout the study, the general adjustment for multiple testing has been applied using Bonferroni correction. Under conservative assumption of 56 independent tests (28 variants analyzed in two independent analyses: OSUMC only and pooled case/control association test) the Bonferroni adjusted p-value threshold in the study was p < 8.9E-04 (0.05/56).

### Imputation

IMPUTE2 was used for imputation^26^ with the 1000 Genomes Phase 3 integrated variant set as the reference panel (October 2014). Imputation was performed jointly for the OSUMC set and MD Anderson control set to ensure uniform information content. Thus, only genotyped variants also present in MD Anderson data were considered for input to imputation. Furthermore, we required that the input variants pass the post-genotyping criteria described above to maximize information content quality. This left all 8 genotyped variants in HERC2/OCA2 locus to be used for inference. Based on these SNPs, an approximately 2MB window encompassing the HERC2/OCA2 locus was imputed. This region contained 6591 variants in the reference panel after filtering to variants common in the European ancestry population (EUR MAF > 0.05). Imputed variants with IMPUTE2 INFO score >0.5 were considered for further analysis resulting in 235 additional variants.

The imputed variants were tested for UM association using logistic regression implemented by the SNPTEST software under an additive model. Genotype uncertainties from imputation were accounted for in the model using genotype dosages. Notably, there were a number of variants comparably significant to our top genotyped associations (Fig. 1 and Supplementary Table 5). To assess if these were independent signals, SNPTEST was used to perform conditional logistic regression based on rs12913832 for all genotyped and imputed HERC2/OCA2 variants again with an additive model and using genotype dosages to account for genotype uncertainty.

## Additional Information

**How to cite this article**: Ferguson, R. *et al*. Genetic markers of pigmentation are novel risk loci for uveal melanoma. *Sci. Rep.* **6**, 31191; doi: 10.1038/srep31191 (2016).

## Supplementary Material

Supplementary Information

## Figures and Tables

**Table 1:** Summary of the top 5 associations with UM risk, passing the Bonferroni adjustments for multiple testing (p < 8.9E-04, see Methods).

SNP	Position (reference assembly b37)	Gene region	Minor allele	Major allele	MAF (OSUMC Cases) N = 272	MAF (OSUMC Controls) N = 735	MAF (GWAS Controls*) N = 1047	OR (95% CI) (OSUMC set)	P-value (OSUMC set)	OR (95% CI) (OSUMC set & GWAS*)	*P*-value (OSUMC set & GWAS*)
rs12913832	chr15:28365617	HERC2/OCA2	A	G	0.16	0.23	0.27	0.562 (0.435–0.731)	1.13E-05	0.529 (0.415–0.673)	8.47E-08
rs1129038	chr15:28356858	HERC2/OCA2	C	T	0.16	0.23	0.27	0.566 (0.438–0.731)	1.34E-05	0.532 (0.419–0.678)	1.19E-07
rs916977	chr15:28513363	HERC2/OCA2	T	C	0.08	0.14	0.17	0.475 (0.339–0.666)	1.55E-05	0.465 (0.339–0.637)	3.04E-07
rs4778138	chr15:28335819	HERC2/OCA2	G	A	0.09	0.14	0.14	0.585 (0.4233–0.808)	1.16E-03	0.591 (0.436–0.802)	5.14E-04
rs12203592	chr 6:396320	IRF4	T	C	0.23	0.19	0.17	1.479 (1.16–1.886)	1.61E-03	1.467 (1.182–1.822)	6.35E-04

The association analysis was performed on 272 UM cases and 1,782 controls, using logistic regression adjusted by age and gender. Minor allele frequencies (MAF) are reported for each case/control population tested and the odds ratios (OR) along with confidence intervals (CI) are reported separately for OSUMC case/control comparison (OSUMC set) and aggregate analysis of OSUMC set and additional controls from a previous GWAS *ascertained at MD Anderson (phs000187.v1.p1)^12^.
