# Supplementary material for: Genetic markers of pigmentation are novel risk loci for uveal melanoma
Source: Sci Rep. 2016 Aug 8;6:31191. doi: 10.1038/srep31191 (PMC4976361; doi:10.1038/srep31191)
Supplement: Supplementary Information [file srep31191-s1.doc]

Genetic markers of pigmentation are novel risk loci for uveal melanoma

Robert Ferguson^1,2,3,*^, Matjaz Vogelsang^1,2,3,*^, Esma Ucisik-Akkaya^1,2,3,*^, Karan Rai^4^, Robert Pilarski^4^, Carlos N. Martinez^1,2,3^, Justin Rendleman^1,2,3^, Esther Kazlow^1,2,3^, Khagay Nagdimov^1,2,3^, Iman Osman^1,3,5,6^, Robert J. Klein^7^, Frederick H. Davidorf^8^, Colleen M. Cebulla^8^, Mohamed H. Abdel-Rahman^4,8**^, Tomas Kirchhoff^1,2,3**^

^1^Perlmutter Cancer Center, New York University School of Medicine, New York, USA

^2^Departments of Population Health and Environmental Medicine, New York University School of Medicine, New York, USA

^3^The Interdisciplinary Melanoma Cooperative Group, New York University School of Medicine, New York, USA

^4^Division of Human Genetics, Department of Internal Medicine, The Ohio State University, Columbus, OH, USA

^5^Department of Medicine, New York University School of Medicine, New York, USA

^6^Ronald O. Perelman, Department of Dermatology, New York University, New York, USA

^7^Department of Genetic and Genomic Sciences, Icahn School of Medicine at Mount Sinai, New York, USA

^8^Havener Eye Institute, Department of Ophthalmology and Visual Science, The Ohio State University, Columbus, OH USA

* Contributed equally to work

** Corresponding author(s)

Corresponding author telephone: 2122639418 email: Tomas.Kirchhoff@nyumc.org

Running title: Novel genetic associations with Uveal melanoma

Keywords: uveal melanoma; pigmentation; GWAS, genetic risk variants

**Supplementary Table 1. Reference studies for the selection of SNPs that are associated with pigmentation and/or skin cancer**

| **SNP ID** | **Gene/**  **Locus** | **Chromosome position** | **GWASs** | **Allele tested** | **Effect size** | **Minimum *P* value** | **Additional GWASs** | **Associated Trait** |
| --- | --- | --- | --- | --- | --- | --- | --- | --- |
| rs1129038 | HERC2/  OCA2 | chr15:28356858 | Amos, C.I. *et al.*[^1^](#_ENREF_1) | C | 0.69 (0.61-0.79)^+^ | 2.58E-08 |  | CM |
| rs12913832 | HERC2  /OCA2 | chr15:28365617 | Amos, C.I. *et al.*[^1^](#_ENREF_1) | A | 0.69 (0.61-0.79) ^+^ | 4.31E-08 | Nan, H. *et al.*[*^2^*](#_ENREF_2)*,* Han, J. *et al*[*^3^*](#_ENREF_3)*.,* Zhang, M.F. *et al*.[^4^](#_ENREF_4) | CM, pigmentation |
| rs4778138 | HERC2/  OCA2 | chr15:28335819 | Guedj, M. *et al.*[*^5^*](#_ENREF_5) | C | 0.8 (0.69-0.92) ^+^ | 6.93.E-03 |  | CM |
| rs916977 | HERC2 | chr15:28513363 | Kayser, M. *et al.*[*^6^*](#_ENREF_6) | G | n/a | 1.19E-43 |  | pigmentation |
| rs8028689 | HERC2 | chr15:28488887 | Han, J. *et al*[*^3^*](#_ENREF_3) | C | -0.25 (0.05)* | 1.50E-07 |  | pigmentation |
| rs11636232 | HERC2 | chr15:28386625 | Han, J.L. *et al.*[^7^](#_ENREF_7) | T | 1.21(0.97-1.51) ^+^ | 9.00E-02 | Han, J. *et al*[*^3^*](#_ENREF_3) | CM, pigmentation |
| rs7495174 | OCA2 | chr15:28344237 | Sulem, P. *et al.*[*^8^*](#_ENREF_8) | G | 4.87 (2.43-9.74) ^+^ | 3.00E-24 |  | pigmentation |
| rs2594935 | OCA2 | chr15:28185037 | Sulem, P. *et al.*[*^9^*](#_ENREF_9) | G | 1.78^++^ | 1.20E-11 |  | pigmentation |
| rs910873 | 20q11.22/  PIGU | chr20:33171771 | Bishop, DT. *et al* [^10^](#_ENREF_10) | A | 1.55 (1.33-1.81) ^+^ | 1.92E-08 | Amos, C.I. *et al.*[*^1^*](#_ENREF_1) | CM |
| rs2284378 | 20q11.22/  RALY | chr20:32588094 | Brown, K.M. *et al.*[^11^](#_ENREF_11) | T | 1.21 (1.1-1.32) ^+^ | 5.69E-05 |  | CM |
| rs258322 | MC1R | chr16:89755902 | Bishop, DT. *et al* [^10^](#_ENREF_10) | A | 1.67 (1.52-1.83) ^+^ | 2.54E-27 | Nan, H. *et al.*[*^12^*](#_ENREF_12) | CM, pigmentation |
| rs4785763 | MC1R | chr16:90066935 | Bishop, DT. *et al* [^10^](#_ENREF_10) | A | 1.36 (1.28-1.45) ^+^ | 5.96E-22 | Han, J.L. *et al.*[*^7^*](#_ENREF_7) | CM |
| rs7023329 | MTAP/  CDKN2A | chr9:21816527 | Bishop, DT. *et al* [^10^](#_ENREF_10) | G | 0.85 (0.80-0.91) ^+^ | 4.03E-07 | Falchi, M. *et al.*[*^13^*](#_ENREF_13) | CM |
| rs2284063 | PLA2G6 | chr22:38544297 | Bishop, DT. *et al* [^10^](#_ENREF_10) | G | 0.83 (0.78-0.88) ^+^ | 2.40E-09 |  | CM |
| rs1393350 | TYR | chr11:89011045 | Bishop, DT. *et al* [^10^](#_ENREF_10) | A | 1.29 (1.21-1.38) ^+^ | 2.41E-14 | Amos, C.I. *et al.*[^1^](#_ENREF_1) | CM |
| rs11018528 | TYR | chr11:88930376 | Nan, H. *et al.*[^12^](#_ENREF_12) | G | 0.13 (0.03)* | 5.90E-06 |  | pigmentation |
| rs1042602 | TYR | chr11:88911695 | Sulem, P. *et al.*[^8^](#_ENREF_8) | A | 1.32 (1.17-1.49) ^+^ | 5.30E-06 |  | pigmentation |
| rs735408 | 14q32.2 | chr14:97097652 | Nan, H. *et al.*[^12^](#_ENREF_12) | A | 0.19 (0.05)* | 2.40E-05 |  | pigmentation |
| rs2424994 | 20q11.22 | chr20:33132916 | Brown, K.M. *et al.*[*^11^*](#_ENREF_11) | T | 1.3 (1.16-1.45) ^+^ | 3.45E-06 |  | CM |
| rs3219090 | 1q42.12 | chr1:226564690 | MacGregor, S. *et al*.[^14^](#_ENREF_14) | G | 0.87^++^ | 9.30E-08 |  | CM |
| rs3750965 | TPCN2 | chr11:68840159 | Sulem, P. *et al.*[*^9^*](#_ENREF_9) | G | 0.607^++^ | 7.50E-09 | Gerstenblith, M.R. *et al*.[^15^](#_ENREF_15) | CM. pigmentation |
| rs896978 | TPCN2 | chr11:68828928 | Sulem, P. *et al.*[*^9^*](#_ENREF_9) | T | 0.617^++^ | 3.80E-08 |  | pigmentation |
| rs12203592 | IRF4 | chr6:396320 | Han, J. *et al*[*^3^*](#_ENREF_3) | T | -0.36 (0.02) * | 7.1E-49 |  | pigmentation |
| rs401681 | TERT-CLPTM1L | chr5:1322086 | Stacey, S.N. *et al.*[*^16^*](#_ENREF_16) | C | 0.86 (0.81-0.91) ^+^ | 3.70E-12 | Rafnar, T. *et al.*[*^17^*](#_ENREF_17) | CM |
| rs1801516 | ATM | chr11:108175461 | Barrett, J.H. *et al*.[^18^](#_ENREF_18) | A | 0.84 (0.79-0.89) ^+^ | 3.4E−09 |  | CM |
| rs1485993 | CCND1 | chr11:69362414 | Barrett, J.H. et al.[^19^](#_ENREF_19) | A | 1.11 (1.04-1.18)^+^ | 1.2E−03 |  | CM |
| rs45430 | MX2 | chr21:42746080 | Barrett, J.H. *et al*.[^18^](#_ENREF_18) | G | 0.88 (0.85-0.92) ^+^ | 2.90E−9 |  | CM |
| rs3754376 | PARP1 | chr1:226561063 | MacGregor, S. *et al*.[^14^](#_ENREF_14) | T | 1.22^++^ | 7.39E-07 |  | CM |
| rs7335046 | UBAC2 | chr13:100041737 | Nan, H. *et al*[*^20^*](#_ENREF_20) | G | 1.26 (1.18-1.34) ^+^ | 2.90E-08 |  | Non-melanoma skin cancer |

**Effect size is presented as a measure of OR with 95% CI ( ^+^), OR where 95% CI were not provided ( ^++^), regression coefficients with standard errors ( ^*^) or n/a when the data was not available.**

**Supplementary Table 2. The demographic and clinical characteristics of UM cases and controls in this study**

| ***CASES*** | |  | ***CONTROLS*** |  |
| --- | --- | --- | --- | --- |
| **Age at pathological diagnosis (years)** | | | **Demographic information on OSUMC controls** | |
| Median (Range) | 59 (18-84) | | **Age (years)** |  |
|  |  | | Median (Range) | 58 (18-94) |
| **Gender** |  | |  |  |
| Male | 133 (49.1%) | | **Gender** |  |
| Female | 139 (50.9%) | | Male | 387 (52.7%) |
|  |  | | Female | 348 (47.3%) |
| **History of cutaneous melanoma (CM)** | | |  |  |
| ***Personal history of CM*** |  | |  |  |
| **No** | **262** | | **Demographic information on Melanoma GWAS controls** | |
| Males | 127 (48.5%) | | **Age (years)** |  |
| Females | 135 (51.5%) | | Median (Range) | 52 (18-87) |
| **Yes** | **10** | |  |  |
| Males | 6 (60.0%) | | **Gender** |  |
| Females | 4 (40.0%) | | Male | 622 (59.4%) |
| ***Family history of CM*** |  | | Female | 425(40.6%) |
| **No** | **232** | |  |  |
| Males | 120 (51.7%) | |  | |
| Females | 112 (48.3%) | |  |  |
| **Yes** | **40** | |  |  |
| Males | 13 (32.5%) | |  |  |
| Females | 27 (67.5%) | |  |  |
| ***No personal or family history of CM*** | **226** | |  |  |
| Males | 116 (51.3%) | |  |  |
| Females | 110 (48.7%) | |  |  |
| ***Personal and family history of CM*** | **4** | |  |  |
| Males | 2 (50.0%) | |  |  |
| Females | 2 (50.0%) | |  |  |
|  |  | |  |  |
| **Status of other cancers** |  | |  |  |
| **No** | **179** | |  |  |
| Males | 87 (48.6%) | |  |  |
| Females | 92 (51.4%) | |  |  |
| **Yes** | **93** | |  |  |
| Males | 46 (49.5%) | |  |  |
| Females | 47 (50.5%) | |  |  |
|  |  | |  |  |
| **UM tumor anatomic site** |  | |  |  |
| Choroidal melanomas | 245 | |  |  |
| Iriociliary melanomas | 16 | |  |  |
| Ciliochoroidal melanomas | 8 | |  |  |
| Others | 3 | |  |  |

**Supplementary Table 3: Association results for all 28 genotyped variants in the study**

| **SNP ID** | **Proxy^+^** | **Locus** | **Minor allele** | **Major allele** | **MAF OSUMC Cases** | **MAF controls (OSUMC controls & GWAS*)** | **OR**  **(95% CI)**  **(OSUMC set & GWAS*)** | **P-value**  **(OSUMC set & GWAS*)** |
| --- | --- | --- | --- | --- | --- | --- | --- | --- |
| rs12913832 |  | HERC2/OCA2 | A | G | 0.16 | 0.27 | 0.531 (0.4168-0.6766) | 8.47E-08 |
| rs1129038 |  | HERC2/OCA2 | C | T | 0.16 | 0.27 | 0.5349 (0.4203-0.6807) | 1.19E-07 |
| rs916977 |  | HERC2/OCA2 | T | C | 0.08 | 0.17 | 0.4666 (0.3406-0.6391) | 3.04E-07 |
| rs4778138 |  | HERC2/OCA2 | G | A | 0.09 | 0.14 | 0.5934 (0.4373-0.8054) | 5.14E-04 |
| rs12203592 |  | IRF4 | T | C | 0.23 | 0.17 | 1.474 (1.188-1.83) | 6.35E-04 |
| rs11636232 |  | HERC2/OCA2 | T | C | 0.45 | 0.38 | 1.303 (1.086-1.563) | 4.61E-03 |
| rs910873 |  | 20q11.22/PIGU | A | G | 0.12 | 0.09 | 1.434 (1.077-1.909) | 1.54E-02 |
| rs7495174 |  | HERC2/OCA2 | G | A | 0.05 | 0.07 | 0.6096 (0.4003-0.9284) | 1.85E-02 |
| rs11018528 |  | TYR | G | A | 0.35 | 0.30 | 1.246 (1.028-1.51) | 2.65E-02 |
| rs1042602 |  | TYR | A | C | 0.33 | 0.38 | 0.8045 (0.6641-0.9746) | 2.81E-02 |
| rs8028689 |  | HERC2/OCA2 | C | T | 0.04 | 0.05 | 0.5971 (0.3742-0.953) | 2.86E-02 |
| rs3750965 |  | TPCN2 | G | A | 0.36 | 0.31 | 1.24 (1.026-1.499) | 2.90E-02 |
| rs2424994 |  | 20q11.22 | T | C | 0.20 | 0.17 | 1.245 (0.992-1.561) | 5.98E-02 |
| rs896978 |  | TPCN2 | A | G | 0.33 | 0.29 | 1.196 (0.9866-1.449) | 7.05E-02 |
| rs2594935 | rs2594934 | HERC2/OCA2 | A | G | 0.25 | 0.29 | 0.8252 (0.6714-1.014) | 7.47E-02 |
| rs401681 | rs380286 | TERT-CLPTM1L | T | C | 0.48 | 0.45 | 1.172 (0.9781-1.405) | 8.59E-02 |
| rs3219090 |  | 1q42.12 | T | C | 0.30 | 0.34 | 0.8462 (0.6952-1.03) | 1.06E-01 |
| rs1393350 |  | TYR | A | G | 0.31 | 0.28 | 1.165 (0.957-1.418) | 1.37E-01 |
| rs7335046 | rs7318947 | UBAC2 | G | C | 0.13 | 0.10 | 1.223 (0.9356-1.598) | 1.49E-01 |
| rs3754376 |  | PARP1 | C | A | 0.30 | 0.34 | 0.8663 (0.7124-1.053) | 1.56E-01 |
| rs735408 | rs17214379 | 14q32.2 | T | C | 0.10 | 0.08 | 1.242 (0.9171-1.681) | 1.62E-01 |
| rs1801516 |  | ATM | A | G | 0.12 | 0.15 | 0.8186 (0.6202-1.08) | 1.80E-01 |
| rs4785763 |  | MC1R | A | C | 0.32 | 0.33 | 0.9305 (0.7664-1.13) | 4.93E-01 |
| rs2284063 | rs6001027 | PLA2G6 | G | A | 0.37 | 0.36 | 1.067 (0.8858-1.286) | 5.03E-01 |
| rs2284378 | rs6120487 | 20q11.22/RALY | T | C | 0.34 | 0.34 | 1.04 (0.859-1.259) | 6.95E-01 |
| rs45430 |  | MX2 | C | T | 0.39 | 0.39 | 0.9644 (0.8018-1.16) | 7.07E-01 |
| rs258322 |  | MC1R | A | G | 0.09 | 0.09 | 0.9758 (0.7144-1.333) | 9.37E-01 |
| rs7023329 |  | MTAP/CDKN2A | A | G | 0.49 | 0.49 | 0.9973 (0.8323-1.195) | 1.00E+00 |

Minor allele frequencies (MAF) are reported for the OSUMC cases and the combined control populations including OSUMC controls and GWAS controls *ascertained at MD Anderson (phs000187.v1.p1)[^1^](#_ENREF_1). The odds ratios (OR) along with confidence intervals (CI) are reported for the aggregate analysis of OSUMC cases and both control populations (OSUMC controls and MD Anderson GWAS). ^+^For SNPs that were not captured in the GWAS controls, an R^2^ = 1 proxy was taken as the target SNP.

**Supplementary Table 4:** **Association results for all 28 genotyped variants in the study when analyzed using different control samples**

|  |  |  | OSUMC Controls | | | MD Anderson controls | | | CGEMS controls | | | Aggregate analysis | | |
| --- | --- | --- | --- | --- | --- | --- | --- | --- | --- | --- | --- | --- | --- | --- |
| SNP | Minor  Allele | Case MAF | Control MAF | OR  (95% CI) | P value | Control MAF | OR  (95% CI) | P value | Control MAF | OR  (95% CI) | P value | Control MAF | OR  (95% CI) | P value |
| rs12913832 | A | 0.16 | 0.25 | 0.562 (0.435-0.727) | 1.13E-05 | 0.27 | 0.4165 (0.324-0.536) | 1.14E-11 | 0.24 | 0.5471 (0.432-0.693) | 5.24E-07 | 0.25 | 0.5658 (0.448-0.715) | 1.83E-06 |
| rs1129038 | C | 0.16 | 0.26 | 0.5657 (0.438-0.731) | 1.34E-05 | 0.27 | 0.4216 (0.328-0.542) | 1.74E-11 | N/A | N/A | N/A | N/A | N/A | N/A |
| rs916977 | T | 0.08 | 0.16 | 0.4752 (0.339-0.666) | 1.55E-05 | 0.17 | 0.3417 (0.247-0.473) | 1.03E-10 | 0.16 | 0.431 (0.315-0.589) | 1.35E-07 | 0.16 | 0.472 (0.347-0.643) | 1.82E-06 |
| rs4778138 | G | 0.09 | 0.15 | 0.5847 (0.423-0.808) | 1.16E-03 | 0.14 | 0.4408 (0.321-0.606) | 4.47E-07 | N/A | N/A | N/A | N/A | N/A | N/A |
| rs12203592 | T | 0.23 | 0.17 | 1.479 (1.16-1.886) | 1.61E-03 | 0.17 | 1.526 (1.206-1.93) | 4.38E-04 | 0.17 | 1.482 (1.207-1.821) | 1.77E-04 | 0.17 | 1.479 (1.209-1.809) | 1.43E-04 |
| rs11636232 | T | 0.45 | 0.38 | 1.313 (1.073-1.607) | 8.11E-03 | 0.38 | 1.425 (1.174-1.731) | 3.44E-04 | 0.40 | 1.258 (1.054-1.501) | 1.10E-02 | 0.39 | 1.244 (1.046-1.479) | 1.37E-02 |
| rs910873 | A | 0.12 | 0.08 | 1.594 (1.151-2.208) | 5.01E-03 | 0.09 | 1.378 (1.004-1.891) | 4.74E-02 | N/A | N/A | N/A | N/A | N/A | N/A |
| rs7495174 | G | 0.05 | 0.08 | 0.5761 (0.367-0.905) | 1.66E-02 | 0.07 | 0.4822 (0.312-0.746) | 1.04E-03 | 0.07 | 0.6028 (0.398-0.913) | 1.68E-02 | 0.07 | 0.6542 (0.435-0.985) | 4.19E-02 |
| rs11018528 | G | 0.35 | 0.30 | 1.263 (1.02-1.564) | 3.20E-02 | 0.30 | 1.201 (0.975-1.479) | 8.46E-02 | 0.29 | 1.317 (1.088-1.594) | 4.69E-03 | 0.29 | 1.291 (1.071-1.555) | 7.22E-03 |
| rs1042602 | A | 0.33 | 0.37 | 0.8084 (0.655-0.998) | 4.82E-02 | 0.38 | 0.8006 (0.65-0.985) | 3.58E-02 | 0.37 | 0.8364 (0.694-1.008) | 6.09E-02 | 0.37 | 0.8256 (0.687-0.992) | 4.12E-02 |
| rs8028689 | C | 0.04 | 0.07 | 0.5262 (0.321-0.863) | 1.09E-02 | 0.05 | 0.454 (0.28-0.735) | 1.32E-03 | 0.05 | 0.5964 (0.377-0.944) | 2.75E-02 | 0.06 | 0.6551 (0.416-1.031) | 6.76E-02 |
|  |  |  |  |  |  |  |  |  |  |  |  |  |  |  |
| rs3750965 | G | 0.36 | 0.31 | 1.237 (1.005-1.521) | 4.43E-02 | 0.31 | 1.286 (1.054-1.57) | 1.34E-02 | 0.31 | 1.227 (1.016-1.482) | 3.37E-02 | 0.31 | 1.239 (1.032-1.486) | 2.14E-02 |
| rs2424994 | T | 0.20 | 0.17 | 1.246 (0.975-1.592) | 7.83E-02 | 0.17 | 1.199 (0.94-1.529) | 1.44E-01 | 0.16 | 1.284 (1.028-1.602) | 2.75E-02 | 0.17 | 1.27 (1.023-1.575) | 3.01E-02 |
| rs896978 | A | 0.33 | 0.30 | 1.189 (0.964-1.466) | 1.05E-01 | 0.29 | 1.274 (1.039-1.561) | 2.01E-02 | 0.29 | 1.206 (0.996-1.46) | 5.57E-02 | 0.29 | 1.207 (1.003-1.453) | 4.60E-02 |
| rs2594935 | A | 0.25 | 0.29 | 0.8175 (0.653-1.024) | 7.94E-02 | 0.29 | 0.7374 (0.593-0.917) | 6.19E-03 | N/A | N/A | N/A | N/A | N/A | N/A |
| rs401681 | T | 0.48 | 0.42 | 1.26 (1.034-1.535) | 2.17E-02 | 0.45 | 1.137 (0.937-1.381) | 1.94E-01 | 0.44 | 1.144 (0.96-1.364) | 1.33E-01 | 0.44 | 1.292 (1.088-1.534) | 3.53E-03 |
| rs3219090 | T | 0.30 | 0.33 | 0.8678 (0.702-1.072) | 1.89E-01 | 0.34 | 0.8476 (0.688-1.044) | 1.20E-01 | 0.32 | 0.9127 (0.752-1.108) | 3.57E-01 | 0.33 | 0.8796 (0.728-1.063) | 1.83E-01 |
| rs1393350 | A | 0.31 | 0.28 | 1.166 (0.936-1.452) | 1.71E-01 | 0.28 | 1.142 (0.921-1.417) | 2.27E-01 | 0.26 | 1.257 (1.034-1.528) | 2.17E-02 | 0.27 | 1.218 (1.007-1.473) | 4.24E-02 |
| rs7335046 | G | 0.13 | 0.12 | 1.091 (0.817-1.456) | 5.56E-01 | 0.10 | 1.306 (0.981-1.738) | 6.71E-02 | 0.10 | 1.324 (1.021-1.716) | 3.42E-02 | 0.11 | 1.278 (0.991-1.649) | 5.88E-02 |
| rs3754376 | C | 0.30 | 0.33 | 0.8895 (0.72-1.099) | 2.78E-01 | 0.34 | 0.8578 (0.697-1.056) | 1.48E-01 | N/A | N/A | N/A | N/A | N/A | N/A |
| rs735408 | T | 0.10 | 0.09 | 1.123 (0.804-1.57) | 4.96E-01 | 0.08 | 1.423 (1.016-1.993) | 4.04E-02 | N/A | N/A | N/A | N/A | N/A | N/A |
| rs1801516 | A | 0.12 | 0.13 | 0.8982 (0.663-1.218) | 4.89E-01 | 0.15 | 0.761 (0.566-1.024) | 7.16E-02 | 0.15 | 0.7876 (0.596-1.041) | 9.35E-02 | 0.14 | 0.7967 (0.607-1.045) | 1.01E-01 |
| rs4785763 | A | 0.32 | 0.33 | 0.9166 (0.742-1.132) | 4.19E-01 | 0.33 | 0.9377 (0.763-1.152) | 5.40E-01 | 0.33 | 0.9314 (0.77-1.126) | 4.63E-01 | 0.33 | 0.9401 (0.781-1.132) | 5.13E-01 |
| rs2284063 | G | 0.37 | 0.35 | 1.095 (0.896-1.339) | 3.76E-01 | 0.36 | 1.057 (0.871-1.282) | 5.76E-01 | 0.35 | 1.096 (0.912-1.318) | 3.28E-01 | 0.36 | 1.074 (0.899-1.283) | 4.32E-01 |
|  |  |  |  |  |  |  |  |  |  |  |  |  |  |  |
| rs2284378 | T | 0.34 | 0.31 | 1.121 (0.912-1.377) | 2.78E-01 | 0.34 | 1.025 (0.833-1.26) | 8.17E-01 | 0.30 | 1.156 (0.959-1.394) | 1.28E-01 | 0.31 | 1.093 (0.911-1.313) | 3.38E-01 |
| rs45430 | C | 0.39 | 0.42 | 0.9109 (0.748-1.11) | 3.54E-01 | 0.39 | 0.9737 (0.803-1.18) | 7.86E-01 | 0.39 | 0.9872 (0.823-1.184) | 8.90E-01 | 0.40 | 0.9753 (0.818-1.164) | 7.81E-01 |
| rs258322 | A | 0.09 | 0.10 | 0.9552 (0.676-1.349) | 7.95E-01 | 0.09 | 1.057 (0.756-1.478) | 7.45E-01 | 0.10 | 0.9115 (0.673-1.234) | 5.49E-01 | 0.10 | 0.9303 (0.689-1.256) | 6.37E-01 |
| rs7023329 | A | 0.49 | 0.49 | 0.9989 (0.82-1.217) | 9.91E-01 | 0.49 | 1.005 (0.831-1.214) | 9.62E-01 | 0.50 | 0.9649 (0.81-1.149) | 6.88E-01 | 0.49 | 0.9789 (0.825-1.162) | 8.07E-01 |

Association was performed by comparing OSUMC cases with three independent control sets: OSUMC controls (genotyped in this study), MD Anderson controls (phs000187.v1.p1) from a recent GWAS on CM[^1^](#_ENREF_1) and control populations from the "Cancer Genetic Markers of Susceptibility" (CGEMS) GWAS data on prostate (phs000207.v1.p1)[^21^](#_ENREF_21) and breast cancer [phs000147.v1.p1] [^22^](#_ENREF_22). The association results, the odds ratios (OR), confidence intervals (CI) and p-values are reported for association tests of OSUMC cases with each control group separately and for the aggregate analysis of OSUMC cases and all three control groups (Aggregate analysis). Minor allele frequencies (MAF) are reported for the OSUMC cases, and respectively for all control populations. "N/A" indicates SNP not present on CGEMS GWAS platform, and hence not tested in CGEMS or Aggregate analysis.

**Supplementary Table 5. Association results for 235 variants with p value <0.05 imputed from 8 genotyped SNPs at HERC2/OCA2 region**

| **SNP** | **Minor**  **allele** | **Major allele** | **INFO** | **MAF (Cases OSUMC)** | **MAF (Controls OSUMC+GWAS)** | **r^2^  with rs12913832** | **OR**  **(95% CI)**  **(OSUMC set & GWAS*)** | **P-value**  **(OSUMC set & GWAS*)** |
| --- | --- | --- | --- | --- | --- | --- | --- | --- |
| rs12912427 | C | T | 0.82 | 0.18 | 0.28 | 0.88 | 1.745 (1.387-2.197) | 7.80E-08 |
| rs1667392 | A | C | 0.85 | 0.18 | 0.28 | 0.85 | 1.756 (1.397-2.206) | 7.98E-08 |
| rs12916300 | C | A | 0.87 | 0.17 | 0.27 | 0.88 | 1.784 (1.412-2.255) | 8.48E-08 |
| rs1614575 | T | G | 0.75 | 0.07 | 0.14 | 0.60 | 2.064 (1.48-2.878) | 1.05E-07 |
| rs12898729 | A | G | 0.79 | 0.21 | 0.30 | 0.88 | 1.656 (1.331-2.061) | 1.25E-07 |
| rs7494942 | G | A | 0.93 | 0.08 | 0.16 | 0.62 | 2.147 (1.566-2.943) | 1.27E-07 |
| rs35299892 | C | T | 0.96 | 0.09 | 0.17 | 0.60 | 2.109 (1.554-2.863) | 1.62E-07 |
| rs1667394 | C | T | 0.98 | 0.09 | 0.17 | 0.60 | 2.129 (1.565-2.895) | 1.71E-07 |
| rs7403279 | C | T | 0.94 | 0.08 | 0.16 | 0.62 | 2.134 (1.555-2.927) | 1.82E-07 |
| rs2525936^✝^ | A | - | 0.92 | 0.09 | 0.16 | 0.52 | 2.023 (1.478-2.769) | 8.51E-07 |
| rs3940272 | T | C | 0.91 | 0.09 | 0.16 | 0.52 | 1.998 (1.461-2.733) | 1.13E-06 |
| rs1635166^✝^ | - | AG | 0.91 | 0.09 | 0.16 | 0.51 | 1.988 (1.455-2.716) | 1.19E-06 |
| rs1667390 | A | G | 0.91 | 0.09 | 0.16 | 0.51 | 1.988 (1.455-2.716) | 1.19E-06 |
| rs201366353 | A | G | 0.90 | 0.09 | 0.16 | 0.52 | 1.985 (1.453-2.712) | 1.20E-06 |
| rs6497293 | T | C | 0.92 | 0.08 | 0.16 | 0.51 | 2.014 (1.469-2.761) | 1.20E-06 |
| rs3935591 | G | A | 0.87 | 0.08 | 0.15 | 0.52 | 1.998 (1.449-2.755) | 1.26E-06 |
| rs8039195 | T | G | 0.92 | 0.08 | 0.16 | 0.51 | 2.011 (1.468-2.757) | 1.28E-06 |
| rs11631797 | A | G | 0.92 | 0.08 | 0.16 | 0.51 | 2.013 (1.468-2.762) | 1.29E-06 |
| rs7170852 | G | T | 0.84 | 0.09 | 0.16 | 0.50 | 1.886 (1.397-2.548) | 1.50E-06 |
| rs1667391 | G | C | 0.66 | 0.06 | 0.11 | 0.27 | 1.948 (1.357-2.798) | 1.74E-06 |
| rs2238289 | T | G | 0.87 | 0.08 | 0.14 | 0.48 | 0.522 (0.376-0.723) | 6.59E-06 |
| rs7496514 | A | G | 0.76 | 0.13 | 0.20 | 0.41 | 0.607 (0.467-0.789) | 1.71E-05 |
| rs4778237 | G | C | 0.75 | 0.13 | 0.20 | 0.40 | 0.608 (0.467-0.791) | 1.81E-05 |
| rs2525964 | A | G | 0.54 | 0.05 | 0.09 | 0.17 | 1.807 (1.209-2.701) | 2.34E-05 |
| rs4778241 | G | A | 0.74 | 0.13 | 0.19 | 0.40 | 1.628 (1.249-2.122) | 2.75E-05 |
| rs62007494 | T | C | 0.73 | 0.05 | 0.09 | 0.25 | 1.894 (1.264-2.839) | 1.57E-04 |
| rs1635168 | C | T | 0.76 | 0.05 | 0.08 | 0.24 | 1.89 (1.247-2.865) | 2.08E-04 |
| rs4932629 | C | T | 0.62 | 0.04 | 0.07 | 0.24 | 0.54 (0.343-0.851) | 2.22E-04 |
| rs12593929 | T | C | 0.75 | 0.04 | 0.08 | 0.27 | 1.93 (1.251-2.977) | 2.38E-04 |
| rs75224447 | A | G | 0.66 | 0.04 | 0.07 | 0.24 | 0.535 (0.34-0.843) | 2.71E-04 |
| rs79286671 | T | C | 0.66 | 0.04 | 0.08 | 0.24 | 0.549 (0.356-0.846) | 2.76E-04 |
| rs8025035 | C | T | 0.78 | 0.04 | 0.08 | 0.26 | 1.922 (1.252-2.95) | 2.94E-04 |
| rs1900758 | T | C | 0.56 | 0.29 | 0.35 | 0.13 | 1.304 (1.071-1.588) | 3.90E-04 |
| rs12595735 | C | T | 0.66 | 0.04 | 0.07 | 0.23 | 0.552 (0.355-0.858) | 4.03E-04 |
| rs7183877 | T | C | 0.69 | 0.04 | 0.07 | 0.24 | 0.522 (0.325-0.837) | 4.15E-04 |
| rs148632780 | G | A | 0.65 | 0.04 | 0.07 | 0.23 | 0.537 (0.336-0.858) | 4.22E-04 |
| rs61756152^✝^ | AT | - | 0.71 | 0.04 | 0.07 | 0.24 | 0.519 (0.324-0.831) | 4.23E-04 |
| rs72714142 | C | A | 0.72 | 0.04 | 0.07 | 0.24 | 0.517 (0.322-0.829) | 4.28E-04 |
| rs72714143 | A | G | 0.72 | 0.04 | 0.07 | 0.24 | 0.517 (0.323-0.83) | 4.29E-04 |
| rs12591531 | C | G | 0.72 | 0.04 | 0.07 | 0.24 | 0.517 (0.322-0.829) | 4.30E-04 |
| rs6497287 | A | G | 0.72 | 0.04 | 0.07 | 0.23 | 0.517 (0.322-0.829) | 4.40E-04 |
| rs76704029^✝^ | - | A | 0.70 | 0.04 | 0.07 | 0.23 | 0.53 (0.335-0.841) | 4.46E-04 |
| rs77572354 | C | T | 0.64 | 0.04 | 0.06 | 0.23 | 0.536 (0.334-0.862) | 4.55E-04 |
| rs7402990 | C | T | 0.68 | 0.03 | 0.06 | 0.23 | 0.527 (0.326-0.85) | 4.91E-04 |
| rs201872292 | C | A | 0.72 | 0.04 | 0.07 | 0.24 | 0.524 (0.328-0.838) | 5.19E-04 |
| rs12438302 | A | G | 0.71 | 0.04 | 0.07 | 0.23 | 0.53 (0.334-0.842) | 5.22E-04 |
| rs4372669 | C | T | 0.72 | 0.04 | 0.07 | 0.24 | 0.524 (0.328-0.838) | 5.26E-04 |
| rs72714147 | A | G | 0.72 | 0.04 | 0.07 | 0.24 | 0.524 (0.327-0.839) | 5.26E-04 |
| rs11637338 | C | G | 0.54 | 0.26 | 0.31 | 0.12 | 1.292 (1.055-1.584) | 5.29E-04 |
| rs11633338 | G | A | 0.54 | 0.26 | 0.31 | 0.12 | 1.292 (1.055-1.584) | 5.29E-04 |
| rs28829928 | A | G | 0.54 | 0.26 | 0.31 | 0.12 | 1.292 (1.054-1.583) | 5.42E-04 |
| rs141685696 | C | T | 0.67 | 0.04 | 0.07 | 0.24 | 0.541 (0.341-0.858) | 5.45E-04 |
| rs2346095 | A | G | 0.66 | 0.03 | 0.06 | 0.23 | 0.525 (0.322-0.859) | 5.52E-04 |
| rs2346094 | T | C | 0.66 | 0.03 | 0.06 | 0.23 | 0.525 (0.322-0.859) | 5.52E-04 |
| rs4932685 | A | G | 0.66 | 0.03 | 0.06 | 0.23 | 0.527 (0.323-0.862) | 5.93E-04 |
| rs2122008 | C | T | 0.52 | 0.26 | 0.31 | 0.12 | 1.285 (1.048-1.575) | 6.05E-04 |
| rs7495521 | A | G | 0.54 | 0.26 | 0.31 | 0.12 | 1.287 (1.05-1.577) | 6.21E-04 |
| rs8033952 | A | C | 0.75 | 0.04 | 0.08 | 0.26 | 1.839 (1.192-2.838) | 6.39E-04 |
| rs11638265 | A | G | 0.57 | 0.26 | 0.31 | 0.14 | 1.297 (1.057-1.593) | 6.54E-04 |
| rs80231418 | T | C | 0.71 | 0.04 | 0.06 | 0.23 | 0.531 (0.331-0.851) | 6.56E-04 |
| rs16950949 | C | G | 0.71 | 0.03 | 0.06 | 0.23 | 0.527 (0.326-0.849) | 6.83E-04 |
| rs7495522 | G | A | 0.53 | 0.26 | 0.31 | 0.12 | 1.284 (1.047-1.573) | 6.92E-04 |
| rs1448488 | A | G | 0.52 | 0.26 | 0.31 | 0.12 | 1.28 (1.044-1.57) | 7.17E-04 |
| rs3214781 | A | G | 0.50 | 0.24 | 0.29 | 0.12 | 1.281 (1.039-1.581) | 7.49E-04 |
| rs12916355 | C | T | 0.52 | 0.26 | 0.31 | 0.12 | 1.273 (1.038-1.562) | 8.94E-04 |
| rs11631735 | C | T | 0.54 | 0.26 | 0.31 | 0.12 | 1.278 (1.042-1.568) | 9.26E-04 |
| rs11632387 | A | G | 0.60 | 0.26 | 0.31 | 0.13 | 1.292 (1.054-1.585) | 9.53E-04 |
| rs12914687^✝^ | GTGT | - | 0.54 | 0.26 | 0.31 | 0.12 | 1.276 (1.041-1.565) | 9.74E-04 |
| rs12903382 | G | C | 0.54 | 0.26 | 0.31 | 0.12 | 1.276 (1.04-1.564) | 9.88E-04 |
| rs12915936^✝^ | - | ACAC | 0.55 | 0.26 | 0.31 | 0.12 | 1.277 (1.043-1.564) | 1.01E-03 |
| rs11636005 | T | C | 0.54 | 0.26 | 0.31 | 0.12 | 1.276 (1.04-1.565) | 1.02E-03 |
| rs34058589 | T | C | 0.52 | 0.25 | 0.30 | 0.12 | 1.27 (1.035-1.56) | 1.03E-03 |
| rs12899295 | A | C | 0.55 | 0.26 | 0.31 | 0.13 | 1.277 (1.042-1.564) | 1.03E-03 |
| rs12910433 | A | G | 0.55 | 0.27 | 0.32 | 0.12 | 1.275 (1.041-1.561) | 1.05E-03 |
| rs8035315 | A | G | 0.56 | 0.26 | 0.31 | 0.13 | 1.278 (1.043-1.565) | 1.05E-03 |
| rs3884517 | T | C | 0.56 | 0.26 | 0.31 | 0.13 | 1.278 (1.043-1.565) | 1.05E-03 |
| rs7162812^✝^ | - | CA | 0.52 | 0.24 | 0.29 | 0.12 | 1.275 (1.034-1.572) | 1.06E-03 |
| rs5811520 | A | G | 0.58 | 0.25 | 0.30 | 0.14 | 1.285 (1.046-1.578) | 1.06E-03 |
| rs9920172 | A | G | 0.60 | 0.26 | 0.31 | 0.13 | 1.286 (1.049-1.577) | 1.13E-03 |
| rs1800411 | G | A | 0.53 | 0.26 | 0.30 | 0.12 | 1.268 (1.033-1.556) | 1.16E-03 |
| rs9788702 | A | G | 0.56 | 0.26 | 0.31 | 0.13 | 1.274 (1.039-1.561) | 1.20E-03 |
| rs11634923 | G | A | 0.53 | 0.26 | 0.30 | 0.12 | 1.267 (1.032-1.555) | 1.21E-03 |
| rs35048626 | A | G | 0.52 | 0.25 | 0.30 | 0.12 | 1.264 (1.029-1.553) | 1.27E-03 |
| rs3216770 | A | G | 0.55 | 0.26 | 0.31 | 0.13 | 1.27 (1.036-1.557) | 1.32E-03 |
| rs12914580 | G | T | 0.61 | 0.26 | 0.31 | 0.13 | 1.283 (1.046-1.574) | 1.37E-03 |
| rs7170451 | G | A | 0.63 | 0.25 | 0.30 | 0.13 | 1.291 (1.051-1.587) | 1.44E-03 |
| rs7182323 | T | C | 0.53 | 0.26 | 0.30 | 0.12 | 1.262 (1.028-1.549) | 1.46E-03 |
| rs4278697 | T | A | 0.52 | 0.26 | 0.30 | 0.12 | 1.261 (1.027-1.548) | 1.47E-03 |
| rs141263240 | A | G | 0.69 | 0.05 | 0.08 | 0.25 | 0.58 (0.386-0.872) | 1.48E-03 |
| rs8042159 | A | C | 0.82 | 0.04 | 0.07 | 0.22 | 0.558 (0.361-0.861) | 1.71E-03 |
| rs7174027 | T | C | 0.77 | 0.09 | 0.13 | 0.21 | 0.647 (0.474-0.883) | 1.74E-03 |
| rs140102770 | A | G | 0.69 | 0.04 | 0.07 | 0.26 | 0.582 (0.371-0.912) | 1.78E-03 |
| rs60727732^✝^ | C | - | 0.53 | 0.02 | 0.03 | 0.03 | 2.038 (1.032-4.022) | 1.82E-03 |
| rs3862443 | A | C | 0.82 | 0.04 | 0.07 | 0.22 | 0.562 (0.363-0.868) | 1.98E-03 |
| rs2346050 | G | T | 0.83 | 0.04 | 0.07 | 0.22 | 0.558 (0.359-0.866) | 2.01E-03 |
| rs7169225 | A | G | 0.64 | 0.25 | 0.30 | 0.14 | 1.281 (1.042-1.574) | 2.08E-03 |
| rs16950987 | C | T | 0.82 | 0.04 | 0.07 | 0.22 | 0.564 (0.365-0.873) | 2.10E-03 |
| rs8041209^✝^ | A | - | 0.82 | 0.04 | 0.07 | 0.23 | 0.56 (0.359-0.873) | 2.22E-03 |
| rs62007492 | A | G | 0.52 | 0.02 | 0.03 | 0.03 | 2.013 (1.012-4.005) | 2.22E-03 |
| rs8023410 | C | A | 0.82 | 0.04 | 0.07 | 0.22 | 0.567 (0.366-0.881) | 2.59E-03 |
| rs58164482 | G | A | 0.82 | 0.04 | 0.07 | 0.22 | 0.563 (0.361-0.879) | 2.64E-03 |
| rs60949565 | T | A | 0.86 | 0.04 | 0.07 | 0.22 | 0.556 (0.355-0.87) | 2.73E-03 |
| rs2525913^✝^ | - | A | 0.85 | 0.04 | 0.07 | 0.22 | 0.557 (0.356-0.871) | 2.74E-03 |
| rs6497289 | G | C | 0.86 | 0.04 | 0.07 | 0.22 | 0.558 (0.358-0.87) | 2.76E-03 |
| rs2016236^✝^ | - | CAA | 0.86 | 0.04 | 0.07 | 0.22 | 0.556 (0.355-0.87) | 2.80E-03 |
| rs77542847 | T | G | 0.60 | 0.05 | 0.07 | 0.20 | 0.635 (0.418-0.966) | 2.80E-03 |
| rs59310062 | A | G | 0.85 | 0.04 | 0.07 | 0.22 | 0.558 (0.358-0.872) | 2.80E-03 |
| rs8036480 | T | G | 0.86 | 0.04 | 0.07 | 0.22 | 0.557 (0.356-0.871) | 2.84E-03 |
| rs73362658 | A | G | 0.85 | 0.04 | 0.07 | 0.22 | 0.558 (0.357-0.872) | 2.84E-03 |
| rs2240201 | T | G | 0.86 | 0.04 | 0.07 | 0.22 | 0.557 (0.357-0.871) | 2.85E-03 |
| rs2240203 | A | G | 0.86 | 0.04 | 0.07 | 0.22 | 0.557 (0.357-0.871) | 2.86E-03 |
| rs201503867 | C | T | 0.86 | 0.04 | 0.07 | 0.22 | 0.559 (0.358-0.872) | 2.87E-03 |
| rs3080838 | T | C | 0.85 | 0.04 | 0.07 | 0.22 | 0.557 (0.355-0.874) | 2.88E-03 |
| rs8030709 | C | G | 0.86 | 0.04 | 0.07 | 0.22 | 0.559 (0.358-0.873) | 2.94E-03 |
| rs2016277 | A | G | 0.86 | 0.04 | 0.07 | 0.22 | 0.557 (0.356-0.873) | 2.96E-03 |
| rs6497292 | T | C | 0.86 | 0.04 | 0.07 | 0.22 | 0.56 (0.358-0.875) | 2.98E-03 |
| rs8036159 | G | A | 0.85 | 0.04 | 0.07 | 0.22 | 0.564 (0.362-0.877) | 2.98E-03 |
| rs13379587 | G | A | 0.85 | 0.04 | 0.07 | 0.22 | 0.559 (0.357-0.875) | 2.99E-03 |
| rs7163496 | A | G | 0.85 | 0.04 | 0.07 | 0.22 | 0.564 (0.362-0.878) | 3.00E-03 |
| rs145071428 | C | T | 0.82 | 0.04 | 0.07 | 0.22 | 0.568 (0.364-0.886) | 3.00E-03 |
| rs62007493^✝^ | - | TA | 0.84 | 0.44 | 0.38 | 0.34 | 1.309 (1.092-1.571) | 3.03E-03 |
| rs2881294 | T | C | 0.84 | 0.04 | 0.07 | 0.22 | 0.567 (0.365-0.882) | 3.04E-03 |
| rs72714118 | T | G | 0.64 | 0.05 | 0.07 | 0.20 | 0.634 (0.42-0.957) | 3.11E-03 |
| rs16950993 | G | C | 0.85 | 0.04 | 0.07 | 0.22 | 0.565 (0.363-0.881) | 3.15E-03 |
| rs60025758 | T | G | 0.86 | 0.04 | 0.07 | 0.22 | 0.561 (0.36-0.875) | 3.15E-03 |
| rs72714121 | G | A | 0.63 | 0.05 | 0.07 | 0.20 | 0.635 (0.421-0.959) | 3.16E-03 |
| rs60556622 | G | A | 0.86 | 0.04 | 0.07 | 0.22 | 0.561 (0.359-0.876) | 3.16E-03 |
| rs12440978 | A | G | 0.85 | 0.08 | 0.12 | 0.19 | 0.639 (0.462-0.884) | 3.28E-03 |
| rs11074326^✝^ | T | - | 0.84 | 0.04 | 0.06 | 0.23 | 0.558 (0.351-0.886) | 3.39E-03 |
| rs72036273 | T | G | 0.74 | 0.04 | 0.06 | 0.23 | 0.577 (0.363-0.918) | 3.47E-03 |
| rs13379995 | T | C | 0.84 | 0.08 | 0.12 | 0.19 | 0.644 (0.466-0.891) | 3.59E-03 |
| rs8030941 | T | C | 0.85 | 0.04 | 0.07 | 0.23 | 0.562 (0.357-0.884) | 3.64E-03 |
| rs5811535 | G | A | 0.53 | 0.02 | 0.03 | 0.03 | 1.934 (1.007-3.716) | 3.66E-03 |
| rs73362608 | G | A | 0.85 | 0.04 | 0.07 | 0.23 | 0.562 (0.357-0.885) | 3.69E-03 |
| rs4778139 | A | T | 0.53 | 0.02 | 0.03 | 0.03 | 1.935 (1.006-3.72) | 3.76E-03 |
| rs8031097 | T | C | 0.86 | 0.04 | 0.07 | 0.23 | 0.563 (0.357-0.887) | 3.88E-03 |
| rs7497759^✝^ | - | T | 0.85 | 0.04 | 0.07 | 0.23 | 0.565 (0.359-0.89) | 3.94E-03 |
| rs7164220 | T | C | 0.73 | 0.08 | 0.12 | 0.21 | 1.509 (1.098-2.072) | 3.96E-03 |
| rs8034699 | A | G | 0.85 | 0.04 | 0.07 | 0.23 | 0.566 (0.359-0.89) | 4.01E-03 |
| rs7165158 | C | T | 0.85 | 0.04 | 0.07 | 0.23 | 0.568 (0.361-0.892) | 4.20E-03 |
| rs4778236 | A | G | 0.83 | 0.08 | 0.12 | 0.18 | 0.652 (0.472-0.899) | 4.26E-03 |
| rs58995112 | A | T | 0.74 | 0.04 | 0.06 | 0.21 | 0.584 (0.367-0.928) | 4.69E-03 |
| rs56839008 | T | G | 0.81 | 0.08 | 0.11 | 0.19 | 0.668 (0.482-0.925) | 7.30E-03 |
| rs1448492 | T | C | 0.69 | 0.26 | 0.30 | 0.12 | 1.234 (1.005-1.514) | 1.04E-02 |
| rs58843292^✝^ | - | G | 0.86 | 0.04 | 0.06 | 0.22 | 0.581 (0.366-0.92) | 1.07E-02 |
| rs7496326 | A | G | 0.90 | 0.04 | 0.07 | 0.23 | 0.58 (0.369-0.912) | 1.12E-02 |
| rs72625134 | A | G | 0.82 | 0.08 | 0.11 | 0.19 | 0.676 (0.486-0.942) | 1.16E-02 |
| rs12593163 | A | G | 0.82 | 0.08 | 0.11 | 0.18 | 0.677 (0.486-0.944) | 1.20E-02 |
| rs57641774 | C | T | 0.89 | 0.04 | 0.06 | 0.22 | 0.595 (0.376-0.944) | 1.31E-02 |
| rs7497270 | A | T | 0.95 | 0.04 | 0.07 | 0.23 | 0.595 (0.387-0.915) | 1.59E-02 |
| rs201840469 | A | G | 0.79 | 0.04 | 0.06 | 0.22 | 0.625 (0.395-0.99) | 1.64E-02 |
| rs72625136^✝^ | T | - | 0.83 | 0.03 | 0.06 | 0.21 | 0.596 (0.37-0.962) | 1.83E-02 |
| rs200844152 | C | A | 0.70 | 0.03 | 0.05 | 0.22 | 0.616 (0.37-1.027) | 1.90E-02 |
| rs10162958 | A | T | 0.96 | 0.03 | 0.06 | 0.22 | 0.592 (0.366-0.957) | 1.94E-02 |
| rs61266109 | C | T | 0.88 | 0.03 | 0.06 | 0.22 | 0.603 (0.372-0.978) | 1.95E-02 |
| rs60107275 | A | T | 0.87 | 0.03 | 0.06 | 0.22 | 0.603 (0.372-0.979) | 1.96E-02 |
| rs12050490 | T | G | 0.93 | 0.04 | 0.06 | 0.22 | 0.603 (0.375-0.97) | 2.07E-02 |
| rs12909057 | A | G | 0.64 | 0.06 | 0.08 | 0.15 | 0.713 (0.49-1.035) | 2.12E-02 |
| rs78699119 | A | G | 0.86 | 0.03 | 0.05 | 0.22 | 0.604 (0.368-0.992) | 2.40E-02 |
| rs367735305^✝^ | C | - | 0.83 | 0.03 | 0.05 | 0.22 | 0.611 (0.373-1) | 2.40E-02 |
| rs77416688^✝^ | - | T | 0.84 | 0.03 | 0.05 | 0.22 | 0.611 (0.373-1.002) | 2.44E-02 |
| rs76925859 | A | C | 0.84 | 0.03 | 0.05 | 0.22 | 0.613 (0.375-1.004) | 2.53E-02 |
| rs2703956 | G | A | 0.67 | 0.32 | 0.36 | 0.06 | 0.846 (0.697-1.025) | 2.54E-02 |
| rs58764974 | G | A | 0.85 | 0.03 | 0.05 | 0.22 | 0.616 (0.374-1.012) | 2.88E-02 |
| rs2525919 | A | G | 0.55 | 0.05 | 0.07 | 0.23 | 0.735 (0.497-1.087) | 2.99E-02 |
| rs79494067 | G | A | 0.83 | 0.03 | 0.05 | 0.23 | 0.619 (0.382-1.004) | 3.00E-02 |
| rs373561014 | C | T | 0.55 | 0.05 | 0.07 | 0.23 | 0.735 (0.497-1.087) | 3.01E-02 |
| rs113713636 | C | T | 0.76 | 0.04 | 0.05 | 0.23 | 0.657 (0.41-1.053) | 3.67E-02 |
| rs75165924 | T | C | 0.79 | 0.04 | 0.05 | 0.23 | 0.649 (0.402-1.048) | 3.71E-02 |
| rs2703948 | A | G | 0.99 | 0.25 | 0.29 | 0.09 | 0.819 (0.667-1.007) | 3.88E-02 |
| rs12904397^✝^ | T | - | 0.88 | 0.03 | 0.05 | 0.22 | 0.632 (0.39-1.023) | 3.98E-02 |
| rs200034854 | A | G | 0.71 | 0.04 | 0.06 | 0.22 | 0.677 (0.429-1.068) | 4.08E-02 |
| rs142477460 | G | A | 0.90 | 0.03 | 0.05 | 0.21 | 0.619 (0.374-1.024) | 4.10E-02 |
| rs2594937 | C | A | 0.99 | 0.25 | 0.29 | 0.09 | 0.822 (0.668-1.01) | 4.11E-02 |
| rs2594936 | T | C | 1.00 | 0.25 | 0.29 | 0.09 | 0.822 (0.669-1.01) | 4.18E-02 |
| rs72625135 | A | G | 0.83 | 0.06 | 0.09 | 0.13 | 0.708 (0.494-1.015) | 4.19E-02 |
| rs2703955 | C | T | 0.92 | 0.26 | 0.30 | 0.08 | 0.83 (0.677-1.017) | 4.28E-02 |
| rs113505002 | T | C | 0.89 | 0.03 | 0.05 | 0.23 | 0.622 (0.373-1.035) | 4.29E-02 |
| rs74400391 | C | A | 0.89 | 0.03 | 0.05 | 0.23 | 0.622 (0.374-1.035) | 4.30E-02 |
| rs78980176 | G | A | 0.89 | 0.03 | 0.05 | 0.23 | 0.622 (0.374-1.036) | 4.30E-02 |
| rs57079108 | T | C | 0.88 | 0.03 | 0.05 | 0.23 | 0.624 (0.376-1.038) | 4.31E-02 |
| rs3932767 | T | C | 0.75 | 0.03 | 0.05 | 0.22 | 0.642 (0.383-1.078) | 4.31E-02 |
| rs12592282 | A | C | 0.89 | 0.03 | 0.05 | 0.23 | 0.623 (0.374-1.036) | 4.35E-02 |
| rs2594933^✝^ | A | - | 0.98 | 0.26 | 0.30 | 0.09 | 0.825 (0.672-1.013) | 4.42E-02 |
| rs77186129 | C | G | 0.86 | 0.03 | 0.05 | 0.23 | 0.633 (0.384-1.043) | 4.45E-02 |
| rs61511707^✝^ | T | - | 0.88 | 0.03 | 0.05 | 0.23 | 0.626 (0.376-1.04) | 4.46E-02 |
| rs76517692^✝^ | A | - | 0.88 | 0.03 | 0.05 | 0.23 | 0.626 (0.376-1.04) | 4.46E-02 |
| rs61756153 | T | C | 0.88 | 0.03 | 0.05 | 0.21 | 0.624 (0.374-1.042) | 4.48E-02 |
| rs80350074 | A | G | 0.88 | 0.03 | 0.05 | 0.22 | 0.625 (0.375-1.041) | 4.50E-02 |
| rs2703953 | T | A | 0.97 | 0.26 | 0.30 | 0.09 | 0.826 (0.673-1.014) | 4.51E-02 |
| rs7495114 | T | C | 0.88 | 0.03 | 0.05 | 0.23 | 0.626 (0.376-1.041) | 4.51E-02 |
| rs76361530 | A | G | 0.89 | 0.03 | 0.05 | 0.23 | 0.627 (0.378-1.04) | 4.54E-02 |
| rs76131506 | A | T | 0.88 | 0.03 | 0.05 | 0.22 | 0.626 (0.376-1.042) | 4.55E-02 |
| rs76512054 | C | A | 0.89 | 0.03 | 0.05 | 0.23 | 0.628 (0.379-1.041) | 4.57E-02 |
| rs79476584 | T | C | 0.88 | 0.03 | 0.05 | 0.23 | 0.628 (0.378-1.042) | 4.57E-02 |
| rs79164713 | T | C | 0.87 | 0.03 | 0.05 | 0.22 | 0.634 (0.384-1.045) | 4.58E-02 |
| rs145688468 | C | G | 0.89 | 0.03 | 0.05 | 0.23 | 0.628 (0.378-1.042) | 4.61E-02 |
| rs7496228 | G | A | 0.89 | 0.03 | 0.05 | 0.22 | 0.628 (0.378-1.043) | 4.65E-02 |
| rs80344102 | A | T | 0.88 | 0.03 | 0.05 | 0.22 | 0.633 (0.384-1.044) | 4.68E-02 |
| rs77093318 | T | C | 0.82 | 0.03 | 0.05 | 0.21 | 0.643 (0.39-1.06) | 4.69E-02 |
| rs199835274 | G | A | 0.88 | 0.03 | 0.05 | 0.22 | 0.634 (0.384-1.046) | 4.69E-02 |
| rs2218679 | G | A | 0.98 | 0.26 | 0.30 | 0.09 | 0.827 (0.674-1.015) | 4.70E-02 |
| rs2240202 | T | C | 0.90 | 0.03 | 0.05 | 0.22 | 0.625 (0.375-1.04) | 4.71E-02 |
| rs2594934 | A | G | 0.97 | 0.26 | 0.29 | 0.09 | 0.827 (0.674-1.016) | 4.72E-02 |
| rs12592363 | A | G | 0.91 | 0.03 | 0.05 | 0.22 | 0.625 (0.376-1.04) | 4.74E-02 |
| rs74950057^✝^ | A | - | 0.90 | 0.03 | 0.05 | 0.21 | 0.626 (0.378-1.039) | 4.74E-02 |
| rs76654715 | G | C | 0.91 | 0.03 | 0.05 | 0.22 | 0.625 (0.376-1.04) | 4.74E-02 |
| rs16950941 | T | A | 0.91 | 0.03 | 0.05 | 0.22 | 0.625 (0.376-1.04) | 4.74E-02 |
| rs16950979 | T | C | 0.90 | 0.03 | 0.05 | 0.22 | 0.625 (0.375-1.041) | 4.75E-02 |
| rs75973130 | G | A | 0.90 | 0.03 | 0.05 | 0.22 | 0.625 (0.376-1.041) | 4.76E-02 |
| rs79087600 | A | G | 0.90 | 0.03 | 0.05 | 0.22 | 0.627 (0.379-1.037) | 4.76E-02 |
| rs16950927 | T | A | 0.90 | 0.03 | 0.05 | 0.22 | 0.626 (0.376-1.041) | 4.76E-02 |
| rs74417197^✝^ | C | - | 0.89 | 0.03 | 0.05 | 0.22 | 0.626 (0.376-1.042) | 4.77E-02 |
| rs61585051 | C | G | 0.88 | 0.03 | 0.05 | 0.22 | 0.631 (0.38-1.049) | 4.78E-02 |
| rs75501824 | G | A | 0.90 | 0.03 | 0.05 | 0.22 | 0.628 (0.379-1.038) | 4.78E-02 |
| rs8182028 | T | C | 0.90 | 0.03 | 0.05 | 0.22 | 0.627 (0.378-1.039) | 4.78E-02 |
| rs74853090 | T | C | 0.88 | 0.03 | 0.05 | 0.22 | 0.634 (0.384-1.047) | 4.79E-02 |
| rs76228202 | C | T | 0.90 | 0.03 | 0.05 | 0.22 | 0.627 (0.379-1.038) | 4.79E-02 |
| rs61368565 | A | G | 0.88 | 0.03 | 0.05 | 0.22 | 0.631 (0.379-1.049) | 4.80E-02 |
| rs181843697 | T | G | 0.86 | 0.03 | 0.05 | 0.22 | 0.642 (0.392-1.052) | 4.81E-02 |
| rs8182077 | A | G | 0.90 | 0.03 | 0.05 | 0.22 | 0.627 (0.378-1.041) | 4.82E-02 |
| rs76653853 | T | G | 0.90 | 0.03 | 0.05 | 0.22 | 0.628 (0.38-1.038) | 4.82E-02 |
| rs12595630 | G | A | 0.91 | 0.03 | 0.05 | 0.22 | 0.627 (0.378-1.04) | 4.86E-02 |
| rs111334430 | C | T | 0.90 | 0.03 | 0.05 | 0.22 | 0.629 (0.38-1.041) | 4.89E-02 |
| rs79097182 | T | C | 0.89 | 0.03 | 0.05 | 0.22 | 0.633 (0.383-1.047) | 4.92E-02 |
| rs2346051^✝^ | A | - | 0.91 | 0.03 | 0.05 | 0.22 | 0.628 (0.378-1.043) | 4.92E-02 |
| rs58395411 | T | C | 0.88 | 0.03 | 0.05 | 0.22 | 0.635 (0.385-1.049) | 4.93E-02 |
| rs2346039 | A | C | 0.88 | 0.03 | 0.05 | 0.22 | 0.635 (0.385-1.049) | 4.93E-02 |
| rs12592730 | G | T | 0.88 | 0.03 | 0.05 | 0.22 | 0.635 (0.384-1.049) | 4.95E-02 |
| rs74940492 | T | C | 0.89 | 0.03 | 0.05 | 0.22 | 0.635 (0.384-1.049) | 4.99E-02 |
| rs2594931 | A | G | 0.92 | 0.26 | 0.29 | 0.09 | 0.834 (0.679-1.023) | 5.00E-02 |
| rs138980831 | A | G | 0.83 | 0.03 | 0.05 | 0.17 | 0.632 (0.375-1.064) | 5.02E-02 |
| rs2594938 | G | T | 0.63 | 0.17 | 0.20 | 0.08 | 0.84 (0.663-1.066) | 5.06E-02 |
| rs2240204 | A | G | 0.91 | 0.03 | 0.05 | 0.22 | 0.631 (0.381-1.046) | 5.08E-02 |
| rs77019455 | C | T | 0.70 | 0.05 | 0.07 | 0.18 | 0.725 (0.49-1.074) | 5.14E-02 |

Minor allele frequencies (MAF) are reported separately for the OSUMC cases and the combined control populations including OSUMC controls and GWAS controls *ascertained at MD Anderson (phs000187.v1.p1)[^1^](#_ENREF_1). The odds ratios (OR) along with confidence intervals (CI) are reported for the aggregate analysis of OSUMC cases and both control populations (OSUMC controls and MD Anderson GWAS). Imputed variants with INFO score >0.5 were considered for the associations analysis as detailed in Methods section.

**^✝^**Short insertion or deletion

**Supplementary references**

1. Amos, C.I. *et al.* Genome-wide association study identifies novel loci predisposing to cutaneous melanoma. *Hum Mol Genet* **20**, 5012-23 (2011).

2. Nan, H. *et al.* Genome-wide association study of tanning phenotype in a population of European ancestry. *J Invest Dermatol* **129**, 2250-7 (2009).

3. Han, J. *et al.* A genome-wide association study identifies novel alleles associated with hair color and skin pigmentation. *PLoS Genet* **4**, e1000074 (2008).

4. Zhang, M.F. *et al.* Genome-wide association studies identify several new loci associated with pigmentation traits and skin cancer risk in European Americans. *Hum Mol Genet* **22**, 2948-2959 (2013).

5. Guedj, M. *et al.* Variants of the MATP/SLC45A2 gene are protective for melanoma in the French population. *Human Mutation* **29**, 1154-1160 (2008).

6. Kayser, M. *et al.* Three genome-wide association studies and a linkage analysis identify HERC2 as a human iris color gene. *Am J Hum Genet* **82**, 411-23 (2008).

7. Han, J.L. *et al.* A Germline Variant in the Interferon Regulatory Factor 4 Gene as a Novel Skin Cancer Risk Locus. *Cancer Research* **71**, 1533-1539 (2011).

8. Sulem, P. *et al.* Genetic determinants of hair, eye and skin pigmentation in Europeans. *Nat Genet* **39**, 1443-52 (2007).

9. Sulem, P. *et al.* Two newly identified genetic determinants of pigmentation in Europeans. *Nature Genetics* **40**, 835-837 (2008).

10. Bishop, D.T. *et al.* Genome-wide association study identifies three loci associated with melanoma risk. *Nature Genetics* **41**, 920-U85 (2009).

11. Brown, K.M. *et al.* Common sequence variants on 20q11.22 confer melanoma susceptibility. *Nature Genetics* **40**, 838-840 (2008).

12. Nan, H. *et al.* Genome-Wide Association Study of Tanning Phenotype in a Population of European Ancestry. *Journal of Investigative Dermatology* **129**, 2250-2257 (2009).

13. Falchi, M. *et al.* Genome-wide association study identifies variants at 9p21 and 22q13 associated with development of cutaneous nevi. *Nature Genetics* **41**, 915-U76 (2009).

14. MacGregor, S. *et al.* Genome-wide association study identifies a new melanoma susceptibility locus at 1q21.3. *Nature Genetics* **43**, 1114-U104 (2011).

15. Gerstenblith, M.R., Shi, J.X. & Landi, M.T. Genome-wide association studies of pigmentation and skin cancer: a review and meta-analysis. *Pigment Cell & Melanoma Research* **23**, 587-606 (2010).

16. Stacey, S.N. *et al.* New common variants affecting susceptibility to basal cell carcinoma. *Nature Genetics* **41**, 909-U69 (2009).

17. Rafnar, T. *et al.* Sequence variants at the TERT-CLPTM1L locus associate with many cancer types. *Nature Genetics* **41**, 221-227 (2009).

18. Barrett, J.H. *et al.* Genome-wide association study identifies three new melanoma susceptibility loci. *Nature Genetics* **43**, 1108-U98 (2011).

19. Barrett, J.H. *et al.* Genome-wide association study identifies three new melanoma susceptibility loci. *Nat Genet* **43**, 1108-13 (2011).

20. Nan, H.M. *et al.* Genome-wide association study identifies novel alleles associated with risk of cutaneous basal cell carcinoma and squamous cell carcinoma. *Human Molecular Genetics* **20**, 3718-3724 (2011).

21. Yeager, M. *et al.* Genome-wide association study of prostate cancer identifies a second risk locus at 8q24. *Nature Genetics* **39**, 645-649 (2007).

22. Hunter, D.J. *et al.* A genome-wide association study identifies alleles in FGFR2 associated with risk of sporadic postmenopausal breast cancer. *Nature Genetics* **39**, 870-874 (2007).
